# Acute cholecystitis: how to avoid subtotal cholecystectomy—preliminary results

**DOI:** 10.1186/s13017-024-00534-x

**Published:** 2024-01-28

**Authors:** Adriana Toro, Martina Rapisarda, Davide Maugeri, Alessandro Terrasi, Luisa Gallo, Luca Ansaloni, Fausto Catena, Isidoro Di Carlo

**Affiliations:** 1 Department of Surgery, University of Catania, Via Santa Sofia 78, 95100 Catania, Italy; 2 Department of Surgical Sciences and Advanced Technologies “G.F. Ingrassia”, University of Catania, Cannizzaro Hospital, General Surgery, Catania, Italy; 3 Department of Clinical, Diagnostic and Pediatric Sciences, University of Pavia, Pavia, Italy; 4 General and Emergency Surgery, Bufalini Hospital, Cesena, Italy

**Keywords:** Acute cholecystitis, Cholecystectomy, Cystic duct, Gallbladder stones

## Abstract

**Background:**

The aim of this manuscript is to illustrate a new method permitting safe cholecystectomy in terms of complications with respect to the common bile duct (CBD).

**Methods:**

The core of this new technique is identification of the continuity of the cystic duct with the infundibulum. The cystic duct can be identified between the inner gallbladder wall and inflamed outer wall.

**Results:**

In the last 2 years, from January 2019 until December 2021, 3 patients have been treated with the reported technique without complications.

**Conclusions:**

Among the various cholecystectomy procedures, this is a new approach that ensures the safety of the structures of Calot’s triangle while providing the advantages gained from total removal of the gallbladder.

## Introduction

Elective surgery for gallbladder stones is usually easy; however, when the indication is acute cholecystitis, with or without stones, the procedure duration, techniques and results are often unpredictable [1].

Many techniques have been described to prevent the various complications of surgery for cholecystitis, particularly damage to the CBD.

Subtotal cholecystectomy is the safest technique to avoid CBD damage; however, this procedure can be associated with some complications [2]. Among others, intraoperative hemorrhage due to inflamed gallbladder wall or postoperative bile leak and subhepatic collection represents the complications of this technique. Furthermore, residual stones in the gallbladder stump can represent a late complication [2].

The aim of this manuscript is to illustrate a new method permitting total cholecystectomy while maintaining safety with respect to CBD.

## Materials and methods

To avoid subtotal cholecystectomy, we recently developed a simple but effective laparoscopic technique to approach and ligate the cystic duct in cases of difficult acute cholecystitis.

The general rules adopted for the laparoscopic cholecystectomy technique have been well standardized over many decades. The patients were positioned supine in the French position, and three ports were used to perform the procedure. The umbilical port was first created through open surgery under direct vision, through which the camera was inserted. After establishing a pneumoperitoneum, two other ports were created to permit a perfect manipulation angle between the tips of the instruments (one 5-mm trocar in the right flank, one 5-mm trocar in the left pararectal).

The main steps of this new technique can be reported in three parts.

1. Isolation of the gallbladder

**Fig. 1:**
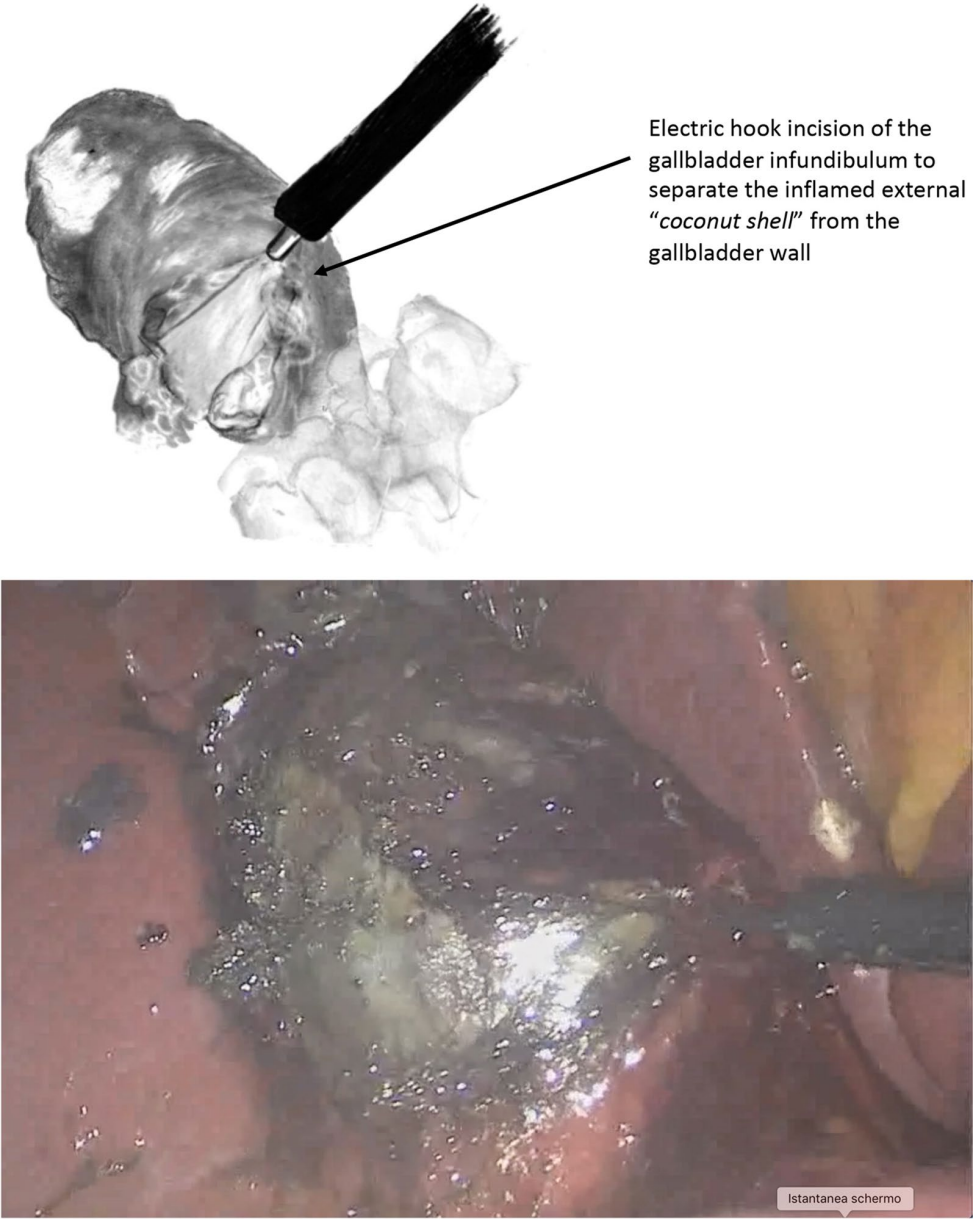
Schematic drawing of the intraoperative view

After releasing the gallbladder from the intraperitoneal adhesions and exposing the entire organ, the upper part of the fundus was identified and lifted up. Three to four centimeters of the inflamed gallbladder wall was then cut using an electrocautery hook (Fig. 1A-B).

An incision was made in a safe zone, under direct vision, at the junction of the infundibulum and the body of the gallbladder. After the incision, the separation between the outer layer (serosa) and inner layer (muscularis layer) was searched. If a space was found between the two layers, tension was created through traction of the inner layer up toward the gallbladder using forceps. No traction was placed on the outer layer, and in this way, it was possible to gradually maneuver it downward and progressively separate it from the inner layer. This maneuver was time intensive and must be done very gently. Step by step, the outer layer was detached from the inner layer, highlighting the cystic duct origin.

2. Identification of cystic duct origin

This clear identification of the confluence between the cystic duct and the body of the gallbladder represents the way to determine whether the procedure was safely completed using the current technique, avoiding damage to the common biliary duct.

**Fig. 2:**
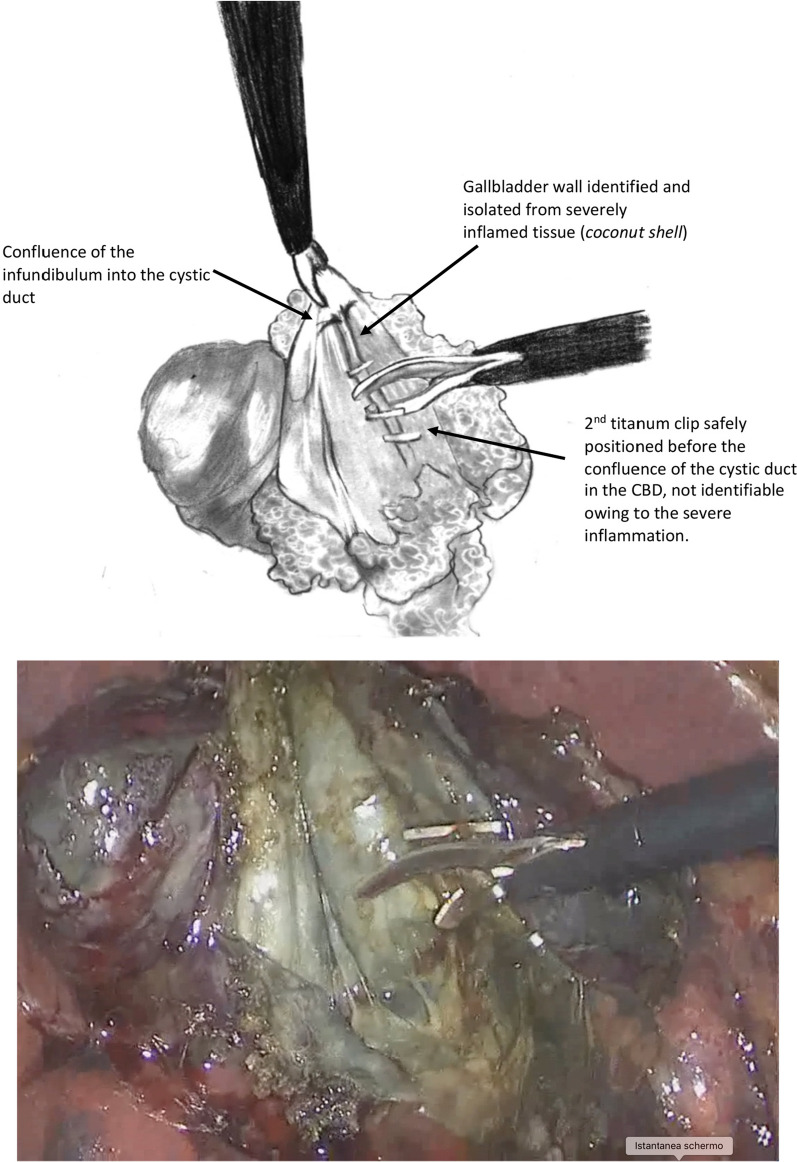
Schematic drawing of the intraoperative view

This maneuver to identify the continuity of the cystic duct with the infundibulum was mandatory (Fig. 2A, B). If this identification was not absolutely clear, another bail-out technique must be applied to complete the procedure safely.

After successful identification, the cystic duct was clipped. It was fundamental to check for a robust inner layer because the mucosa only can be so thin, and the clip positioned on the cystic duct can be released, causing complications.

3. Finale step of the technique.

The gallbladder can then be lifted and turned over, leaving the unidentified CBD untouched, and cholecystectomy can be carefully performed as usual. The inflamed posterior wall remained attached to the gallbladder bed of the liver. At the end of the procedure, a scrupulous check for biliary leakage and hemostasis was performed. A suction drainage tube was placed.

## Results

In the last 2 years, from January 2019 until December 2021, 3 patients have been treated with the reported technique. Two females and 1 male with a mean age of 50 years were diagnosed by CT scan with stage II acute cholecystitis according to the Tokyo guidelines and were operated on within three days of symptom onset. The abdominal drainage tube was left in place for 1 day, and following negative US findings, the patients were discharged on the 2nd postoperative day. No complications have been reported. Pathological examination of the specimen never showed only the mucosa layer, and the mucosa was associated with muscular layers of the gallbladder wall.

## Discussion

Total cholecystectomy represents the best method to cure all gallbladder diseases, and the Strasberg indications surely represent the basic guidelines to prevent the various complications associated with difficult cholecystectomies [3].

Four types of subtotal cholecystectomies have been proposed [2]. In types A and B, part of the posterior wall remains attached to the liver; however, in type A, the remaining gallbladder stump remains open, whereas in type B, it is closed. Types C and D include resection of both the anterior and posterior gallbladder walls; however, in type C, the pouch is closed, and drains are not routinely used, whereas in type D, the stump remains open.

Despite the many techniques used, ligature of the cystic duct, when possible, remains the gold standard for cholecystectomy. When subtotal cholecystectomy is performed, complications can occur; for example, some studies have shown a higher incidence of postcholecystectomy syndrome, cystic duct leakage, biliary event recurrence, biliary fistula, readmissions and reinterventions [4–6].

Many articles describe the possibility of Calot visualization [7]. In case of severe inflammation of Calot, this maneuver is not easy and should be avoided to prevent severe damages [8] or open conversion [9]. Furthermore, the visualization of Calot triangle is very hazardous in case of severe inflammation and a cautious behavior could be recommended [10].

The first step of the present technique is the most important not only to avoid complications but also to understand if the technique can be finalized or not. As the approach is very high, far from infundibulum, the only risk in this stage is represented by wall perforation. In case of minimal leakage, the procedure can reach the end. On the opposite in case of massive bile leakage in our opinion is better to convert with subtotal cholecystectomy, laparoscopic [2] or open [11]. In the presence of massive adhesion and difficulty to separate the layers, the subtotal cholecystectomy has to be adopted without procrastination.

The bleeding that can affect this procedure is not relevant as for common total cholecystectomy. This is due to the fact that the majority of anterior vessels can be managed easily. The posterior ones between the external layer and the liver, in this technique, are leaved in place with external layer.

Safe dissection of Calot's triangle to obtain a sufficient surgical view and minimize vascular or biliary injury is not easy. Our technique differs from that of Calot exposure because the cystic duct is identified from inside the inner gallbladder wall and externally from the inflamed outer wall, similar to dissecting a *coconut shell.* In this way, the continuity between the cystic duct and the infundibulum ensures that no other structures can be injured. The thickness of the gallbladder wall due to inflammation makes Calot exposure very challenging; in contrast, with our technique, the differentiation between the layers of the gallbladder permits safe identification of the cystic duct. This differentiation represents the safety of the presented technique, which is based on the identification of the junction of the cystic duct origin from the infundibulum after separation of the outer and inner layers of the inflamed gallbladder wall. This visualization represents the fundamental step of the technique because in this way, all catastrophic complications can be avoided, such as immediate direct clipping of the common bile duct or unnecessary traction and late complications such as secondary biliary cirrhosis [12]. This technique is reproducible and can represent another useful option that can be useful in cases of acute cholecystic complications that make it difficult to operate. This procedure requires care from the surgeon side, but the advantage is that the affected zone of confluence of the cyst into the common bile duct is not approached from outside the peritoneum that covers these zones, avoiding the related risks.

Among the various cholecystectomy procedures, this is a new approach that ensures the safety of the structures of Calot’s triangle while providing the advantages gained from total removal of the gallbladder.

To the best of our knowledge, there are no similar techniques in the literature. A mucosectomy has been described by Gagner M. et al. [13] with the intent of skeletonizing the wall of the gallbladder and excluding the gallbladder from the bile duct, but the manuscript reports different indications.

## Conclusion

This technique represents a new technique in the armamentarium of surgeons in cases of very difficult cholecystitis. All patients who undergo surgery for acute cholecystitis could be treated with this technique, when possible. This approach should be tentatively attempted. If the technique can be completed, a safe cholecystectomy can be achieved, avoiding all the complications of subtotal cholecystectomy. This technique may fail if separating the two layers of the gallbladder wall is impossible, as a safe dissection plane to safely demonstrate the conjunction of the cystic duct with the infundibulum of the gallbladder would not be obtained.

The technique can be very useful, but surgeons should remember that is not mandatory, and in cases of difficulties, a bail-out procedure is needed for the benefit of both the patients and surgeons.

## Data Availability

The datasets used and/or analyzed during the current study are available from the corresponding author upon reasonable request.

## References

[1] Franklin ME, George J, Russek K (2010). Needlescopic cholecystectomy. Surg Technol Int.

[2] Toro A, Teodoro M, Khan M, Schembari E, Di Saverio S, Catena F, Di Carlo I (2021). Subtotal cholecystectomy for difficult acute cholecystitis: how to finalize safely by laparoscopy-a systematic review. World J Emerg Surg.

[3] Mayumi T, Okamoto K, Takada T, Strasberg SM, Solomkin JS, Schlossberg D, Yamamoto M (2018). Management bundles for acute cholangitis and cholecystitis. J Hepatobiliary Pancreat Sci.

[4] Walsh RM, Ponsky JL, Dumot J (2002). Retained gallbladder/cystic duct remnant calculi as a cause of postcholecystectomy pain. Surg Endosc.

[5] Brunt LM, Deziel DJ, Telem DA, Strasberg SM, Aggarwal R, Asbun H, Bonjer J, McDonald M, Alseidi A, Ujiki M, Riall TS, Hammill C, Moulton CA, Pucher PH, Parks RW, Ansari MT, Connor S, Dirks RC, Anderson B, Altieri MS, Tsamalaidze L, Stefanidis D (2020). Safe cholecystectomy multi-society practice guideline and state-of-the-art consensus conference on prevention of bile duct injury during cholecystectomy. Ann Surg.

[6] Strasberg SM, Pucci MJ, Brunt LM, Deziel DJ (2016). Subtotal cholecystectomy-"fenestrating" vs "reconstituting" subtypes and the prevention of bile duct injury: definition of the optimal procedure in difficult operative conditions. J Am Coll Surg.

[7] Manatakis DK, Antonopoulou MI, Tasis N, Agalianos C, Tsouknidas I, Korkolis DP, Dervenis C (2023). Critical view of safety in laparoscopic cholecystectomy: a systematic review of current evidence and future perspectives. World J Surg.

[8] Honda G, Iwanaga T, Kurata M, Watanabe F, Satoh H, Iwasaki K (2009). The critical view of safety in laparoscopic cholecystectomy is optimized by exposing the inner layer of the subserosal layer. J Hepatobiliary Pancreat Surg.

[9] Ota A, Kano N, Kusanagi H, Yamada S, Garg A (2003). Techniques for difficult cases of laparoscopic cholecystectomy. J Hepatobiliary Pancreat Surg.

[10] van de Graaf FW, Zaïmi I, Stassen LPS, Lange JF (2018). Safe laparoscopic cholecystectomy: a systematic review of bile duct injury prevention. Int J Surg.

[11] Di Carlo I, Pulvirenti E, Toro A, Corsale G (2009). Modified subtotal cholecystectomy: results of a laparotomy procedure during the laparoscopic era. World J Surg.

[12] Chiche L, Guieu M, Bachellier P, Suc B, Soubrane O, Boudjema K, Navarro F, Adam R, Vaillant JC, Salame E, Heyd B, Truant S, Adam JP, Laurent C (2022). Liver transplantation for iatrogenic bile duct injury during cholecystectomy: a French retrospective multicenter study. HPB (Oxford).

[13] Gagner M, Blanco R, Rossi RL (1993). Ultrasonic mucosectomy of the gallbladder. A histological analysis. HPB Surg.

